# Energy Harvesting Sources, Storage Devices and System Topologies for Environmental Wireless Sensor Networks: A Review

**DOI:** 10.3390/s18082446

**Published:** 2018-07-27

**Authors:** Michal Prauzek, Jaromir Konecny, Monika Borova, Karolina Janosova, Jakub Hlavica, Petr Musilek

**Affiliations:** 1 Faculty of Computer Science, VSB Technical University of Ostrava, 708 33 Ostrava, Czech Republic; jaromir.konecny@vsb.cz (J.K.); monika.borova@vsb.cz (M.B.); karolina.janosova@vsb.cz (K.J.); jakub.hlavica@vsb.cz (J.H.); 2 Department of Electrical and Computer Engineering, University of Alberta, Edmonton, AB T6G 1H9, Canada; petr.musilek@ualberta.ca

**Keywords:** environmental monitoring, maintenance-free nodes, sensor network, IoT, energy harvesting, solar power, wind power, thermal power, energy storage, supercapacitor, batteries, network topology

## Abstract

The operational efficiency of remote environmental wireless sensor networks (EWSNs) has improved tremendously with the advent of Internet of Things (IoT) technologies over the past few years. EWSNs require elaborate device composition and advanced control to attain long-term operation with minimal maintenance. This article is focused on power supplies that provide energy to run the wireless sensor nodes in environmental applications. In this context, EWSNs have two distinct features that set them apart from monitoring systems in other application domains. They are often deployed in remote areas, preventing the use of mains power and precluding regular visits to exchange batteries. At the same time, their surroundings usually provide opportunities to harvest ambient energy and use it to (partially) power the sensor nodes. This review provides a comprehensive account of energy harvesting sources, energy storage devices, and corresponding topologies of energy harvesting systems, focusing on studies published within the last 10 years. Current trends and future directions in these areas are also covered.

## 1. Introduction

**Figure 1:**
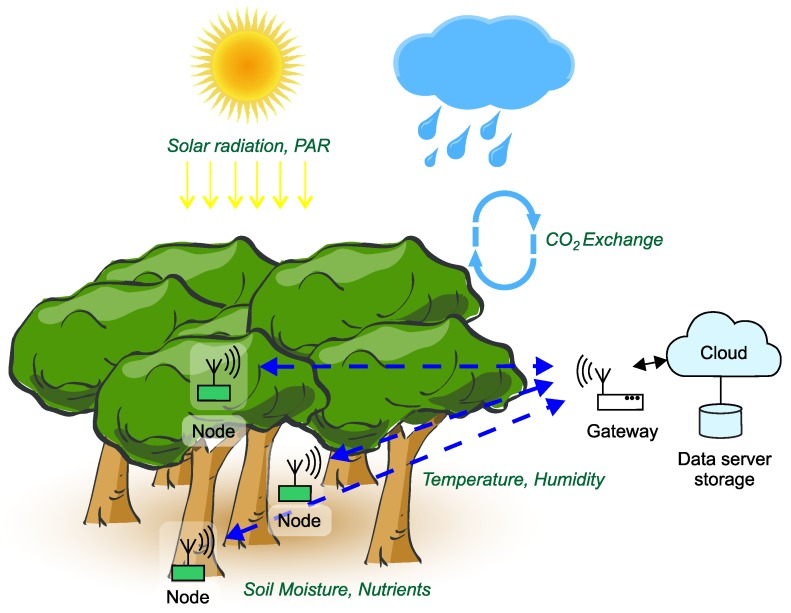
A monitored terrestrial ecosystem with its main processes and characteristics.

Environmental wireless sensor networks (EWSNs) are typically used to obtain information about environments that is needed for decision making. In this context, “environmental monitoring” may refer to observation of indoor or outdoor spaces, whether natural or man-made. Relevant approaches include remote sensing using aircraft and satellites, laboratory analysis of field-collected samples, and in-situ monitoring using sensor devices and networks. They monitor chemical, biological or population-related parameters of the environments under surveillance [1]. As an example, Figure 1 illustrates a suite of variables monitored in the context of terrestrial ecosystems. Such environments represent a wide range of environmental energy regimes, from rainy seasons in tropical rain-forests to polar nights in Arctic deserts.

The development of environmental monitoring nodes still poses many research challenges. EWSNs are often deployed far from inhabited centers, and thus without access to mains electricity [2]. This is at the root of the primary challenge: the selection of an appropriate topology and suitable operating strategies [1] that ensure the energy efficiency of the nodes [3]. In some applications, energy efficient design can be supplemented with energy-for-data trade-off [4], allowing the extension of node operational life, albeit at the cost of sub-optimal data collection rates.

The selection of an appropriate power supply is a crucial step in EWSN design. Primary (non-rechargeable) batteries are typically the first option for powering a field-installed EWSN. Energy harvesting devices offer an alternative (or supplement) to primary batteries that can greatly extend the lifetime of EWSN devices. Harvesting sources are often combined with primary batteries and other energy storage devices [1].

The remaining sections of this review article are organized as follows. Section 2 describes suitable technologies for powering energy-independent sensor nodes. The possibilities of storing harvested energy for future use are detailed in Section 3. Section 4 describes high-level models of EWSN power supplies. Section 5 identifies current challenges and elaborates topics for future research in the area of EWSNs. The final Section 6 brings major conclusions. A taxonomy of references covered in this review is provided in Table A1 of Appendix A.

## 2. Energy Sources

In general, the goal of energy harvesting is to convert energy from one form to another that can be used to power electronic devices. When implemented in environmental monitoring nodes, it can directly extract ambient energy from the environment under surveillance and use it to power the nodes of the EWSN, improving their performance and/or extending their lifetime. Outdoor environments offer plenty of opportunities to take advantage of the elements naturally present in the surroundings, such as wind or sun. However, there are also other types of energy, such as radio frequency (RF) signals resulting from human activities that be scavenged and used to power the nodes. This section provides a systematic overview of common harvesting sources suitable for environmental monitoring nodes, and their comparison.

Energy harvesting sources can be categorized as ambient or external [5]. The ambient sources are accessible within an environment without any external energy supply. They include, for example, radio frequency (RF), solar, thermal, flow-based, and vibration energy harvesting sources [6]. The external sources emit energy to the environment, with the intent for this energy to be harvested by the nodes. Examples include human or mechanical sources, which are not suitable for environmental monitoring purposes.

### 2.1. Solar-Based Sources

One of the most commonly used sources of energy for harvesting is the sun [7]—an affordable and clean energy source. Solar energy is uncontrollable, but it can be predicted through daily and seasonal patterns [8].

Solar power is transformed into electrical power using photovoltaic cells. The amount of output power generated by a cell depends on the intensity of light as well as cell size and effectiveness, according to the photovoltaic principle [9]. To increase the output power, multiple cells are usually combined into modules, also known as solar panels. Individual solar cell maximum output power can also be improved by advanced design methods, such as cross-layer optimization [10]. Photovoltaic cells can be classified according to the type of material they are made of: mono-crystalline (with efficiency of 15–24%), polycrystalline (with efficiency of 14–20.4%) and thin-film (with efficiency of 8–13.2%) [11,12].

A photovoltaic system is capable of producing power ranging from μW to MW depending on its area. A typical value of power density considered when designing energy harvesting for embedded systems is 15 mW/cm${}^{2}$ [13]. Energy obtained from solar panels is usually stored in (rechargeable) batteries or supercapacitors [5,14].

**Figure 2:**
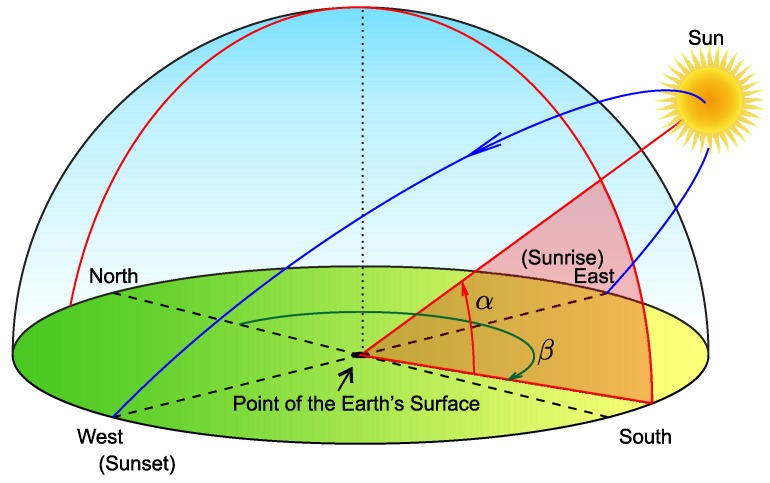
Illustration of the position of the solar disk for maximum efficiency, adapted from [15].

Correct position of the solar panel is important to achieve its maximum possible efficiency. As shown in Figure 2, the location of the sun in the sky relative to a location on the surface of the Earth can be defined by the solar altitude α (the angle between sun’s position and the horizontal plane of the Earth’s surface) and by the solar azimuth β (the angle between a vertical plane incorporating the solar disk and a line running due north) [15].

The global horizontal irradiance GHI (${\mathrm{Wm}}^{-2}$) consists of direct normal irradiation (DNI), coming directly from the solar disk under the solar zenith angle Θ, and diffuse horizontal radiation (DHI), which is scattered by molecules and particles in the atmosphere:

(1) GHI=DNI·cosΘ+DHI.

**Figure 3:**
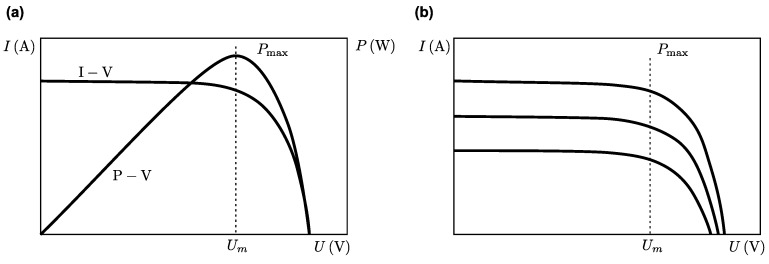
Current-Voltage (I-V) and Power-Voltage (P-V) characteristic curve of solar cells: (**a**) constant irradiance; (**b**) variable irradiance, adapted from [19].

As illustrated in Figure 3, the solar cell is not a constant voltage and current source. The output power of the cell depends on the sunlight intensity and the ambient temperature. The maximum power point tracking (MPPT) controllers ensure that solar cells always operate around the maximum power point $P_{\max}$ under diverse irradiance and temperature conditions, and variable load characteristics [12,16,17,18,19].

The utilization of available solar energy can be improved using numerous modeling and forecasting approaches. Harvested energy prediction using environmental shadow detection can be used to adapt sensor nodes’ scheduling plans according to energy availability and residual battery levels [20]. A systematic approach to power subsystem capacity planning for solar energy harvesting embedded systems is described in [21]. It is based on a modified astronomical model to approximate the harvestable energy and calculate the required battery capacity. The inputs of this model are the latitude of the deployment site, orientation and inclination angles of the solar panel, and expected meteorological and environmental conditions. A system comprised of a solar panel, lithium battery, and MPPT control circuit can take full advantage of solar energy to extend the life of the rechargeable battery [22,23].

### 2.2. Thermal-Based Sources

Thermal energy can be converted to electricity by thermoelectric transducers depending on spatial variations in temperature or pyroelectric transducers depending on temporal variations in temperature.

Thermoelectric transducers and generators are based on the Seebeck effect [24]. They are composed of several pairs of *p*- and *n*-type semiconductor blocks ordered in parallel and connected electrically in series. The open circuit voltage of a thermoelectric element depends on the temperature difference (T) between the hot and cold sides, and on material properties (Seebeck coefficients).

Thermoelectric generators have low efficiency (only about 5–6%). When placed in a stable heated environment, the thermoelectric system will stop generating power after a while when both plates achieve the same temperature [5,11,25].

Pyroelectric converters can be realized as an alternating current source in combination with a capacitor. The pyroelectric current depends on the speed of the temperature change of the pyroelectric material:

(2) $$i_{p}\left(t\right)=p^{\prime }A\frac{dT}{dt},$$

where *p* is the component of the pyroelectric coefficient vector, *A* is the surface area of the electrode, and dT/dt designates the temperature change over time.

Thermal energy harvesting systems have been used in many outdoor applications. A prototype device for harvesting thermoelectric energy from asphalt pavement roadways collects heat energy from the pavement surface and transfers it to the thermoelectric generators embedded at the edge of the pavement [26]. A 64 × 64 mm system can generate about 10 mW of electrical power permanently over a period of 8 h.

### 2.3. Wind-Based Sources

Outdoor WSN applications can use energy extracted from the wind. This type of energy harvesting converts kinetic energy into electricity using turbines, rotors, and the principles of electromagnetic induction [5,25].

A common approach is to use a small wind turbine. Turbine selection starts with the determination of a suitable airfoil, which depends on the Reynolds number. For an average chord length of 2 cm and for a wind speed of 6 m/s, the Reynolds number is around 10,000. An analysis of horizontal axis wind turbines with low Reynolds number is described in [27], along with the implementation of wind turbines for expected power coefficient and maximum power transfer with a small generator. According to the analysis, increasing the number of blades provides a higher power coefficient because the increase in torque compensates the decrease in angular velocity. By comparison, a three-bladed turbine with 10 cm radius produces more power with a relatively low efficiency, while a 5.5 cm radius four-bladed turbine has lower power production but higher average efficiency. The six-bladed system is reported to be a good compromise between efficiency, delivered power, and size [27].

Another option is the use of a wind flutter generator [28] based on the aeroelastic flutter effect. This device consists of aeroelastic ribbon, magnets, and an electromagnetic transducer. In comparison with turbine generators, the windbelt represents a direct conversion generator and has no rotor, bearings, or gears. The prototype of this device was tested using a wind tunnel.

A wind generator capable of supplying up to 10 mW using a 6.3 cm diameter turbine and a 16 km/h wind speed is described in [29]. Another system [30] uses a turbine with 3 cm radius to produce 7.86 mW at 3.62 ${\mathrm{ms}}^{-1}$ wind speeds. The proposed WSN architecture processes the weather forecast from Internet and transmits it to the end devices. This allows the sensor nodes to better plan their energy use [31]. Baranov et al. [32] use a hybrid power supply combining solar and wind energy sources to power nodes that monitor carbon monoxide levels in urban areas and outdoor industrial facilities.

### 2.4. RF-Based Sources

RF-based energy harvesting uses the transmission of radio waves ranging from 3 kHz to 300 GHz, and their subsequent conversion to direct current. This can be accomplished using a single-stage or multistage converter, depending on the requirements of power, voltage, or efficiency. The amount of harvestable power is given by the source power, antenna gain, and by the distance from the RF source. The conversion efficiency ranges from 50% to 75%.

There are two models for RF power harvesting and following communication with other sensor nodes: single radio or two radios. In the latter case, one radio receives the RF signal and the other is used for communication. In the other case, a single radio serves both purposes, reducing the complexity of harvesting and communication software.

One major limitation of RF power sources is that the strength of the signal decreases with distance, leaving only very low power levels available for harvesting [11]. Due to the increasing penetration of wireless communication and broadcasting infrastructure (analog/digital TV, AM/FM radio, Wi-Fi networks, etc.), the energy density of ambient RF is steadily increasing, especially in urban environments [33].

The main advantage of RF-based energy harvesting in comparison with solar, thermal, or flow-based sources is its availability in indoor environments [34]. Potential applications include smart homes, health monitoring, and environmental monitoring (pollution, agriculture) [5].

A prototype of an embedded microcontroller-enabled sensor platform powered by an ambient ultrahigh-frequency digital TV signal (512–566 MHz) has been proposed by Kim et al. [33]. The authors also presented a dual-band (915 MHz/2.45 GHz) ambient energy harvester. The work of Dinesh Kumar and Hemalatha [35] showed that a microstrip antenna could harvest energy from a 2.4 GHz Wi-Fi signal. The addition of a ground signal ground probe embedded with a matching circuit could attain a total power conversion efficiency of 3.8% for −40 dBm input power and 18.2% for −30 dBm input power at 2.35 GHz [36]. An embedded wireless energy-harvesting prototype has been used to power and sustain a 16-bit embedded microcontroller, scavenging wireless power from a TV broadcaster located over 6.3 km away [37].

### 2.5. Comparison of Harvesting Sources

The comparison of main energy harvesting sources provided in (Table 1) shows that wind provides the highest power density and nominal power. However, wind harvesting systems are often large, and wind is not always available. Solar energy is not permanently available either, but has better predictability due to its diurnal and seasonal periodicity. Ambient RF sources are available throughout the day, but have very low power density and their strength rapidly decreases with increasing distance from the source. Thermal-based energy sources are unpredictable, but controllable. The value of power density or output range varies depending on the type of use in individual sources cited in table [11].

## 3. Energy Storage

The term energy storage describes technology to convert energy from a form that is difficult to store (e.g., electrical energy) to a storable form (e.g., electrochemical). The stored energy can then be converted back into a directly usable form. There are various types of energy storage with different properties, such as capacity, power, and charge/discharge rates. The choice of a particular technology depends on the application requirements. In the context of environmental monitoring, energy storage units must satisfy a specific set of requirements related to their small size, adequate capacity, and low environmental impact.

The following types of storage devices are typically used to power environmental monitoring sensor nodes:

- Primary or secondary batteries;
- Supercapacitors;
- Hybrid combinations of supercapacitors and rechargeable batteries.

The energy storage subsystem is a very important component of a sensor node, greatly affecting its overall efficiency. The choice of energy storage technology also affects the size, cost, and operating life of the node [11].

### 3.1. Batteries

The choice of battery can be approached from many perspectives. The most important factors affecting the choice are application requirements (e.g., need of quick charging/discharging, lifetime, cycling, size, weight). Batteries typically serve not just to supply the system with energy, but also to efficiently store energy harvested from the environment. This way, energy can be stored for times when it cannot be directly extracted from the surroundings.

Important battery specifications include storage technology, energy density, internal resistance, depth of discharge, self-discharge, and tolerance to overcharging. From the application perspective, it is important to clearly specify operating conditions and choose appropriate battery devices to avoid operational problems. One example is battery selection for different climatic conditions (e.g., tropical vs. arctic regions) [39].

Batteries can be primary or secondary. Primary batteries are non-rechargeable and can be one of the choices for EWSNs. They have many advantages, including high capacity and temperature stability. Their main disadvantage is the need for periodic maintenance and replacement at the end of life. Secondary batteries are rechargeable. However, their number of charge/recharge cycles is still limited by cycling capacity.

Non-rechargeable dry cell batteries can be divided to alkaline and acidic categories. The alkaline batteries have slightly better performance, while the acidic batteries are more dependable and less expensive.

Most batteries, except lithium-ion, do not perform well in cold temperatures due to the increase of their internal resistance, which leads to a loss of capacity. The opposite holds true for their operation at elevated temperatures, but at the cost of a significant shortening of their service life or even permanent damage. The estimated overall efficiency of battery storage is in the range from 60% to 80%, depending on the operational cycle and the electrochemistry type within the batteries [40].

The energy density (Wh/kg) indicates the maximum density of the stored energy in the battery per unit of mass, and differs for individual battery chemistries. The battery capacity is the amount of energy that can stored in the cell at the full charge. The lifetime of most electrochemical batteries is on the order of hundreds to thousands of charging/discharging cycles. During this time, the battery capacity gradually decreases because of the chemical corrosion of its electrodes. The lifetime is greatly influenced by charging and discharging, as well as by the operating temperature [41]. The basic parameters of selected battery types are summarized in Table 2.

The ambient temperature of the battery plays an important role in estimating its real life. A typical battery achieves nominal characteristics at temperatures around 20 ${}^{\circ }$C. Any significant deviations from this temperature may result in shorter battery life and more frequent battery charges [42]. Rechargeable batteries can also be combined with supercapacitors to form a hybrid storage system that extends operational time.

Lead-Acid batteries are most commonly used for medium-sized devices. Their advantages include low cost, high reliability, and high efficiency. However, they have low cycling capacity and poor performance in extreme conditions [48]. NiCd batteries have long lifetime, fast charging, and vibration resistance. Their main disadvantage is low capacity. NiMH batteries have an improved capacity, but are also less toxic and thus more suitable for environmental monitoring applications. The lithium-ion batteries have high efficiency, power density, and cell voltage. However, their high cost along with a tendency to cause fires when exposed to moisture limits their use [48]. Alkaline MnO${}_{2}$ batteries have the lowest self-discharge rate [49].

### 3.2. Supercapacitors

Supercapacitors are characterized by high power density compared to batteries and common capacitors. They are constructed either as electrochemical double layer capacitors (EDLCs) or pseudocapacitors [50]. The EDLC (ultra-capacitor) works on the electrochemical principle. The electric charge is situated between electrodes with high surface area and thinner electrolytic dielectrics. Their maximum operating voltage is given by the breakdown parameters of the dielectric material. Their rated voltage includes a safety margin to prevent electrolyte decomposition and subsequent short circuit [11,25]. Pseudocapacitors have lower power density than EDLC devices, but provide higher specific capacitance and energy density [50]. They use a redox reaction that occurs on an electrode, generates charges, and transfers them across a layer.

Compared to rechargeable batteries, supercapacitors have several advantages:

- Large number of charge/discharge cycles without a significant decrease of performance and storage capacity: around 500,000 to 1,000,000 cycles, depending on the manufacturer [11,51].
- High charge/discharge efficiency (up to 98%) and fast charging process [11].
- Wide range of operating temperatures between −40 ${}^{\circ }$C and +65 ${}^{\circ }$C for both EDLC supercapacitors and pseudocapacitors [51]. Some sources report an even wider range −55 ${}^{\circ }$C and +85 ${}^{\circ }$C.

However, the use of supercapacitors is often affected by self-discharge—a problem related to the terminal voltage of the energy stored in the element [52]. The magnitude of the problem depends on device capacity, but also differs among manufacturers and even among individual production batches. Several studies have reported self-discharge rates ranging from 50–60% per month [11,51] to 5.9% or even 11% per day [51,53]. If not addressed, leakage can significantly decrease the operational time of powered devices [54]. It can be compensated for by using fast recharging. Together with regularly available and efficiently exploited ambient energy, fast recharging can offer an operating lifetime of a estimated 20 years [55].

Initially, it was assumed that all energy losses in supercapacitors are due to leakage. However, a study by Merret et al. [52] expanded this view in two important ways. First, they observed that voltage drops considerably faster with shorter charge times. Digital electronic circuits of a typical WSN can only operate down to a certain voltage threshold (typically in the range of 1–2 V), and hence the longer charge times can provide a usable voltage for considerably longer periods. Second, this effect is accentuated when the supercapacitor is also loaded. Further tests with sensor nodes duty cycled at 0.1% confirmed that the supercapacitor-powered devices can operate considerably longer when charged for longer periods. Basic parameters of supercapacitors are summarized in Table 3.

### 3.3. State-of-Charge Estimation

Accurate estimation of the state-of-charge (SOC) is an important task for any energy storage application. It is a complex task because there are many phenomena that cause energy storage aging. They include loss of charge acceptance of the active material on the electrodes, changes in physical properties of the electrolyte, and corrosion of the current conductors [56]. For this reason, another measurable parameter describing the physical state of a battery is usually also monitored: the state-of-health (SOH) [57].

For effective operation of EWSN power supplies, the estimation of SOC is important to properly set-up their operational parameters (e.g., measurement and transmission periods) [2]. Common SOC estimation methods have been described in several dedicated reviews [56,58,59]. Approaches suitable for EWSN-type embedded devices are summarized in Table 4.

Coulomb counting (also known as ampere-hour counting) is the most common primary battery management circuit in modern electronic embedded designs with energy storage. The corresponding electronic circuit, called the “gas gauge” [59], is based on voltage measurement on a small (5–50 mΩ) resistor connected in series with a load. It monitors charging and discharging current and then determines SOC through calculations [60]. An alternative technique measures the open circuit voltage of the battery. It is more cost-effective, but suffers from low accuracy and low dynamic range [56]. Impedance spectroscopy (including measurement of internal resistance) is a common method to measure electrochemical processes that can also be used to determine SOC and SOH [56]. The most reliable method to determine the remaining capacity of an energy storage device is the discharge test. This test is not practical, as it is time-consuming and can only be performed offline because the system operation must be interrupted [56]. As such, it is only useful to estimate the storage capacity at the beginning of the energy storage life-cycle.

### 3.4. New Trends in Energy Storage

Most new trends in storage technology are connected to the development of new materials. The use of graphene-based materials in Li-ion, Li–S, Li–O${}_{2}$, Na-ion batteries, and in supercapacitors has been evaluated in [61]. The results have shown that the use of graphene in selected devices can greatly improve their performance. Song et al. [62] demonstrated high areal capacity by using a novel cathode (enhanced by nitrogen-doped carbon sulfur nanocomposite) in LiS battery technology. The properties of Li-ion batteries have been improved (high specific capacity of 2250 mAh, compared to 740 mAh of standard Li-ion batteries) using red phosphorus for anodes, as reported in [63].

Bichat et al. [64] developed new materials for building symmetric capacitors from seaweed carbons. They exhibited an excellent cycle life for voltage values up to 1.6 V, and can be used to manufacture environment-friendly components with high energy density. A new eutectic ionic liquid mixture based on imides was proposed in [65] for use as electrolyte in supercapacitor applications with a large range of operating temperatures. The electrolyte exhibits excellent thermal properties, and the electrochemical performance of the device is characterized by a wide electrochemical window of 3.5 V, maintaining an excellent double-layer capacitive behavior and great cycling stability. An asymmetric capacitor that offers two times the power of its symmetrical counterpart has been proposed in [66]. It is based on activated carbon in an organic electrolyte, and is thus extremely promising for the development of environmentally friendly systems.

## 4. Topologies of Energy Harvesting Systems

The ultimate goal of a typical wireless sensor node is to collect data of interest ad infinitum. In order to operate indefinitely, a system cannot consume, on average, more power than a harvested source can provide. Otherwise, if the consumption exceeds the production, the system will eventually deplete its energy stockpile and stop working due to the empty energy reservoir and absence of environmental energy. This leads to undesirable system performance.

Kansal et al. [67] take a step further and present a theory of energy-neutral operation. Similar work has been presented in [68,69,70,71]. The main improvement is that the non-idealities of energy storage devices are considered, yielding a sounder theory. In [72], the sources and the consumers are modeled using the same mathematical model. This new model can also be considered as a generalization of [67]. Since the reasoning is similar for both theories, only the most relevant substance is presented here.

There are three main topologies for energy harvesting systems: autonomous, hybrid autonomous, and battery-supplemented. Depending on the configuration, energy management strategies with different design goals are required [73].

### 4.1. Autonomous Harvesting Systems

Autonomous harvesting systems fully satisfy their energy needs from ambient sources, without batteries [74,75]. Autonomous systems can only operate when the energy source is available, but their lifetime and performance are not limited by storage inefficiencies (e.g., round-trip efficiency, self-discharge, and aging). These systems are inherently governed by the so-called energy neutrality principle, since they can never consume more energy than their harvesting device can deliver. They must be designed for maximum performance (i.e., to perform at the maximum level that can be supported in a given harvesting environment) [72]. To support these design goals, autonomous harvesting systems should employ prediction algorithms that give estimates of future available energy over time [76]. A proper energy management strategy should allow such a system to achieve the desired utility within a variable energy environment [67].

**Figure 4:**
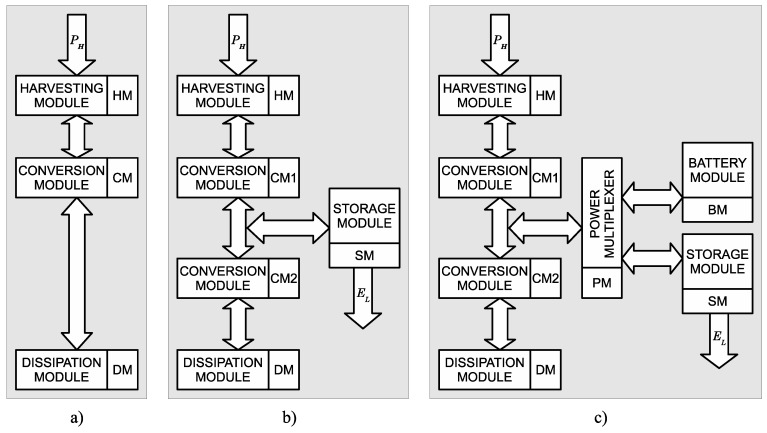
Topologies of harvesting systems: (**a**) autonomous harvesting system; (**b**) autonomous hybrid harvesting system; (**c**) battery-supplemented harvesting system.

The structure of an autonomous harvesting system is shown in Figure 4a. This is the simplest case, which consists of three major modules [77]: an energy harvesting module (HM), an energy converter module (CM), and an energy dissipation module (DM). The main disadvantage of this type of harvesting system is that if the load consumes less energy than what is available from the environment, the excess energy will be lost. The HM is the only source of energy in the system. There is no energy buffer. The CM (typically implemented by a DC/DC converter) supplies energy directly to the DM (load). The energy harvested in the time interval $\left(t_{1},t_{2}\right)$ can be expressed as follows:

(3) $$E_{H}\left(t_{1},t_{2}\right)=\int_{t_{1}}^{t_{2}}P_{H}\left(t\right)dt.$$

The distribution of power over time $P_{H}\left(t\right)$ is variable, and characterization of the stochastic source depends on the type of HM. The main advantage of this system is the need for only one CM. This module may also implement an MPPT algorithm for a specific type of HM. The efficiency of energy transfer from the harvesting module to the DM is described by

(4) $$E_{D}\left(t_{1},t_{2}\right)<=\int_{t_{1}}^{t_{2}}P_{H}\left(t\right)\cdot \eta \left(u,i\right)dt,$$

where η(u,i) is a nonlinear efficiency coefficient of the CM. It depends on the time distribution of power with respect to the voltage, u(t), and current, i(t), output of the HM. This coefficient can be asymptotically approximated by a continuous or piecewise linear function. Alternatively, it can be modeled by a nonlinear graph of measured efficiency values.

### 4.2. Autonomous Hybrid Harvesting Systems

Autonomous hybrid harvesting systems are the most common type of energy harvesting system. They have an energy reservoir implemented using a secondary battery or ultracapacitor [78,79]. The harvesting device collects energy for system operation and the recharging of storage [13]. This arrangement can dramatically increase the operational lifetime of the system. With proper energy management, this topology can achieve 0% dead time operation. The battery and the energy harvesting device must be sized so that they satisfy the energy needs of the system, possibly using the energy-neutrality principle [67]. The system can sometimes consume more energy than the harvesting source provides (using battery reserves), but the production/consumption rates have to be balanced over the long run.

An autonomous hybrid harvesting system with energy leakage is described in Figure 4b. The system contains the three modules of the autonomous system (HM, CM, and DM), and an energy storage module (SM) [77]. $P_{H}\left(t\right)$ is a continuous bounded function of a continuously varying parameter *t*. $P_{H}\left(t\right)$ is an appropriate source in the hybrid harvesting system if and only if for any finite real time interval, $t_{2}-t_{1}$, it satisfies the following two inequalities:

(5) $$\int_{t_{1}}^{t_{2}}P_{H}\left(t\right)dt\geq \rho \left(t_{2}-t_{1}\right)-\sigma_{1},$$

(6) $$\int_{t_{1}}^{t_{2}}P_{H}\left(t\right)dt\leq \rho \left(t_{2}-t_{1}\right)+\sigma_{2},$$

where $P_{H}$ is harvested power from the environment where the integral of $P_{H}$ in the closed interval of $T=t_{2}-t_{1}$ represents total energy, and $\sigma_{1}$ and $\sigma_{2}$ are energy storage capacity coefficients. $P_{H}\left(t\right)$ models the power output of an energy source at time *t*. Variable ρ represents power (in Watts), while constants $\sigma_{1}$ and $\sigma_{2}$ correspond to energy (in Joules) [67].

A method to determine $\sigma_{1}$ and $\sigma_{2}$ from practical measurements is provided in [67]. A device utilizes its energy source fully and can operate forever if it is supplied by a so-called $\left(\rho ,\sigma_{1},\sigma_{2}\right)$-source of energy and operates at a constant power. The energy storage module is usually a secondary battery or supercapacitor with a limited capacity, $E_{C}$. When stored energy $E_{S}$ reaches $E_{C}$, the incoming harvested energy overflows the energy storage. In addition, one can define two energy threshold levels. A low-energy threshold, $E_{\theta_{L}}$, indicates the limit below which the device goes into a sleep mode, and the amount of remaining stored energy is reserved to maintain the contents of volatile memory [77]. The high-energy threshold, $E_{\theta_{H}}$, indicates the limit above which the device reverts back to the normal operating mode.

(7) $$E_{\theta_{L}}\leq E_{S}\left(t\right)\leq E_{C},$$

where

(8) $$E_{C}\geq \sigma_{1}+\sigma_{2}.$$

An autonomous hybrid harvesting system with an ideal energy storage device can be described as follows:

(9) $$\int_{t_{1}}^{t_{2}}\frac{P_{D}\left(t\right)}{\eta_{2}\left(t\right)}dt\leq \int_{t_{1}}^{t_{2}}P_{H}\left(t\right).\eta_{1}\left(t\right)dt+E_{S},$$

where $P_{D}$ is the dissipation power, $P_{H}$ is the harvested power, and $E_{S}$ is the stored energy. Coefficients $\eta_{1}\left(t\right)$ and $\eta_{2}\left(t\right)$ express the efficiencies of the energy converter modules CM1 and CM2, respectively. These efficiencies depend on the operating points of the HM ($\eta_{1}$) and DM ($\eta_{2}$), which implies their nonlinear dependency on voltage and current.

Real storage devices leak energy, $P_{L}$, that can be described as follows:

(10) $$\int_{t_{1}}^{t_{2}}\frac{P_{D}\left(t\right)}{\eta_{2}\left(t\right)}dt\leq \int_{t_{1}}^{t_{2}}P_{H}\left(t\right).\eta_{1}\left(t\right)dt+E_{S}-\int_{t_{1}}^{t_{2}}P_{L}\left(t\right)dt,$$

where (10) differs from (9) by the last term.

### 4.3. Battery-Supplemented Harvesting Systems

Battery-supplemented harvesting systems usually have a battery as the main source of energy and a harvesting device that plays an important, but secondary, role. The goal of energy management in such systems is to limit battery energy usage and to increase the system’s lifetime (e.g., by making external recharging or replacement of batteries less frequent) [72]. This system can use primary or secondary batteries. Harvested energy can directly or indirectly power the load or its specific parts. An example can be found in [80]. This approach greatly increases system reliability and allows data acquisition, processing, and transfer. As long as the primary batteries have some useful charge left, the system can continue to operate in situations when secondary storage is depleted and environmental energy is not available for harvest.

A battery-supplemented harvesting system is shown in Figure 4c. In addition to the components of an autonomous hybrid harvesting system (HM, CM, DM, and SM), there is a primary (non-chargeable) battery module (BM) and a power multiplexer (PM). The energy function is similar to (10), but includes an additional term:

(11) $$\int_{t_{1}}^{t_{2}}\frac{P_{D}\left(t\right)}{\eta_{2}\left(t\right)}dt\leq \int_{t_{1}}^{t_{2}}P_{H}\left(t\right).\eta_{1}\left(t\right)dt+E_{S}+E_{B}-\int_{t_{1}}^{t_{2}}P_{L}\left(t\right)dt,$$

where $E_{B}$ represents energy stored in the primary batteries.

## 5. Research Challenges

**Figure 5:**
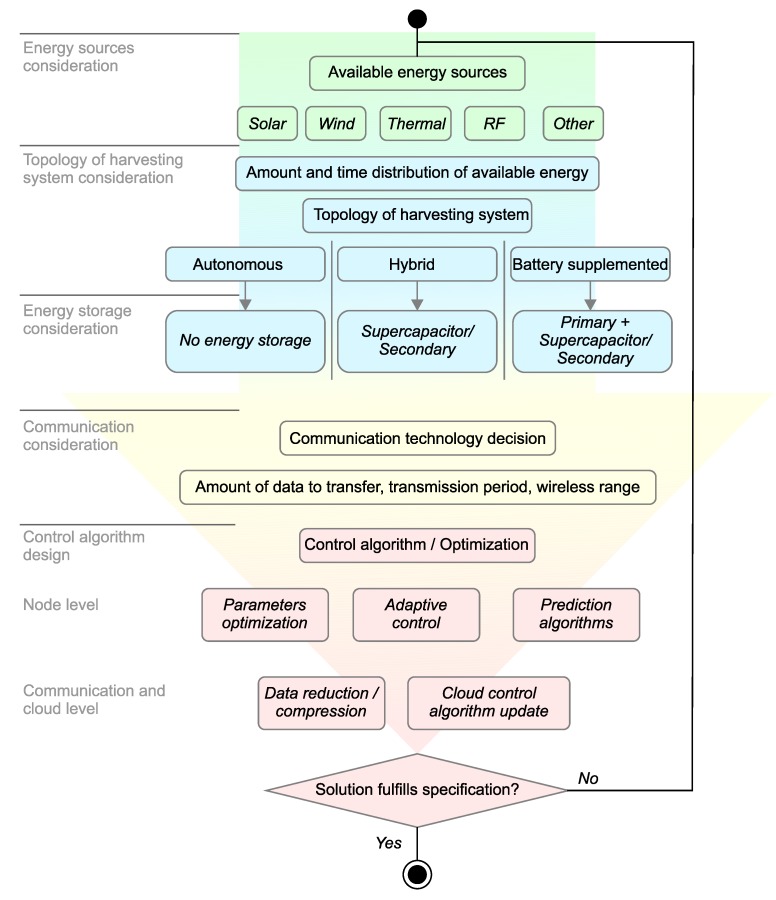
Flowchart of environmental wireless sensor network (EWSN) design process.

It is likely that several techniques described in the previous sections need to be combined to achieve an effective EWSN design. These techniques include the selection of energy harvesting sources and methods, the choice of storage technology, the application of appropriate data acquisition and processing methods, the implementation of suitable communication techniques, and the design of a control algorithm. Selected techniques are then combined into a complex strategy suitable for a specific-purpose EWSN. Figure 5 shows a flowchart representing the various steps of EWSN design, and implementation options available at each stage.

At the beginning of the EWSN design process, an analysis of the available energy sources should be performed. One or more energy sources must be selected and composed into the energy harvesting module. The selection of an appropriate harvesting system topology (autonomous, hybrid, or battery-supplemented) is driven by the amount of harvestable energy and the time distribution of its availability. In the case of hybrid and battery-supplemented topologies, an energy storage technology must also be added to the design. The next step is the selection of a suitable communication technology. This decision depends primarily on the amount of data to transfer, transmission period, and required wireless range. There may also be the deployment site-specific criteria such as local availability of gateways or wireless network coverage. The final step is the design of the control algorithm. On the node level, different algorithms can be utilized, including adaptive and predictive control techniques. At the communication or cloud level, data reduction/compression and cloud control algorithm update can be employed.

**Figure 6:**
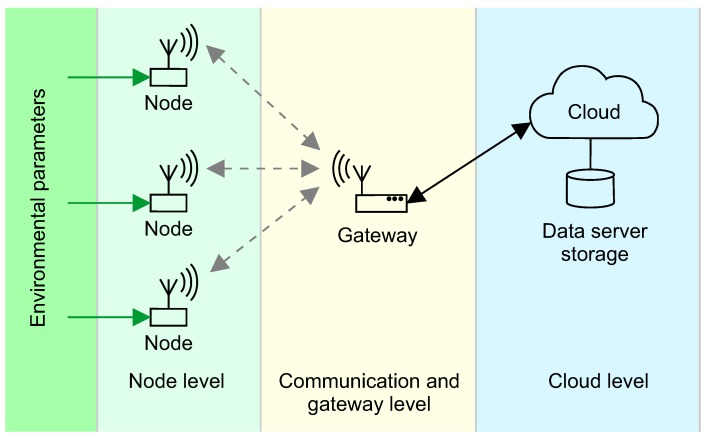
Research challenges: node level, communication and gateway level, and cloud level.

The future research in the area of energy-independent environmental monitoring will likely concentrate on several topics found at various levels of monitoring system infrastructure, as depicted in Figure 6: a number of research challenges can be found at node, communication, and cloud levels.

### 5.1. Single Node Perspective

**Figure 7:**
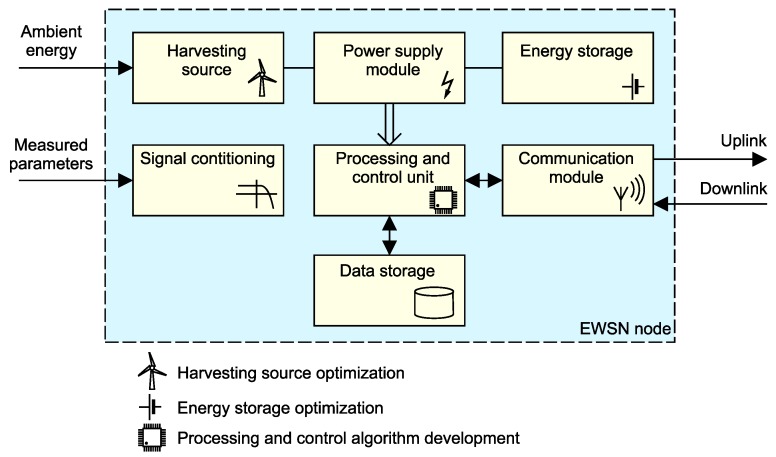
Node block diagram with highlighted research challenges: harvesting source and energy storage optimization, algorithms for processing and control unit.

The main research challenges at the level of a single monitoring node perspective are shown in Figure 7. The EWSN node hardware design is more of an engineering task. On the other hand, the selection or optimization process for parameters of the energy harvesting source or energy storage unit represents a complex research problem whose solution may lower manufacturing cost and increase node reliability.

These parameters are related to a particular deployment environment. For instance, measurement nodes deployed in tropical regions will have different requirements compared to those to be deployed in the Arctic [81,82].

Specific research challenges concentrate on the transfer of energy from harvesting source to storage. They range from the design of energy transducers, through the optimization of power transfer (such as maximum power-point tracking, MPPT), to the development of devices converting electrical energy to a different form matching the selected energy storage. Corresponding research goals focus on increasing the efficiency of power conversion. In the area of solar- and thermal-based energy transducers, there have been many advances related to material research with the same goal. Kinetic energy converters for wind harvesting are miniaturized to fit the form factor of embedded devices. RF-based harvesting is very promising for EWSN applications due to their ability to obtain energy from wireless transmitters combined with the continuous reduction of node power consumption.

Another research challenge considers a measurement node and its focus on operating cycle. A previous study [83] provided evidence that monitoring nodes can be operated using a set of static parameters optimized off-line. These parameters typically include measurement rate and data transmission cycle equidistantly distributed in the time domain. However, such static approaches are constrained by energy incoming from a harvesting source: when the energy source shows significant seasonal dependency, then the EWSN can in certain cases seize normal operation.

Difficulties due to diurnal or seasonal variability can be addressed using advanced control algorithms based on soft-computing or artificial intelligence methods. One example is the use of a fuzzy controller for the adaptive control of sensing and transmission parameters in measurement nodes [4]. Alternative approaches to such systems include, for instance, reinforcement learning [84].

Advanced control approaches can also be combined with various optimization methods, such as differential evolution, particle-swarm optimization, or genetic algorithms. Our previous study [2] develops an optimization method used to tune the parameters of the fuzzy controller, introduces a harvesting-aware predictive controller, and describes the selection of the optimal prediction horizon for harvested energy.

### 5.2. Network and Cloud Perspective

Other research challenges can be identified at the network level. When communication with a measurement node is established, the data from that node can be sent to a cloud as an uplink data stream. Many communication technologies described in this section can also setup a downlink communication channel, which allows a specific data frame to be sent from the cloud to the node itself.

**Figure 8:**
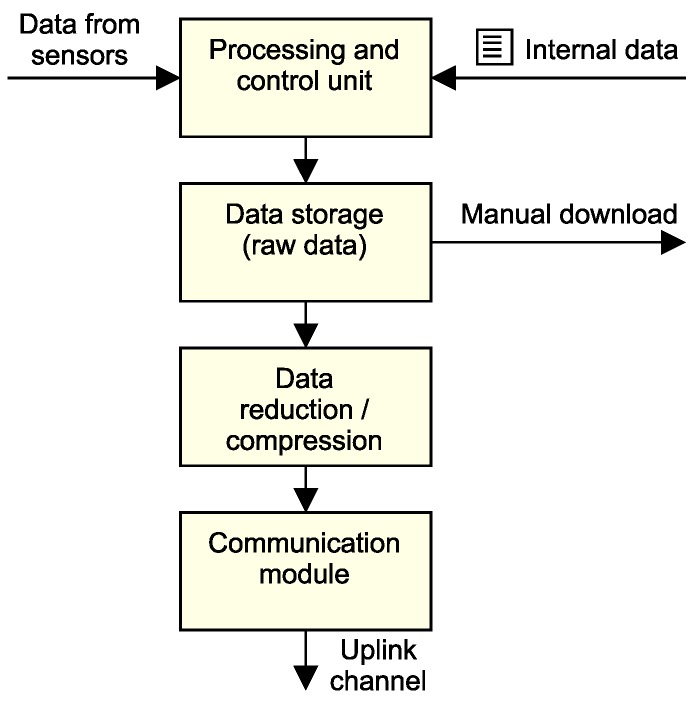
Data handling in an uplink data channel.

The most important research objective at the network level concentrates on reducing the volume of data that needs to be sent in both uplink and downlink directions. The underlying communication technology usually strictly limits the amount of data transferred within a specific window of time [85]. Therefore, methods for data compression or reduction can be added to the development pipeline, as shown in Figure 8.

The first scenario describes data reduction by an internal compression algorithm, where the node sends only compressed data. The raw dataset is not stored on the measurement node. In the second scenario, the measurement node sends a reduced data set and the raw data file is stored on the node. The raw data is then available for manual download during maintenance of the device. This configuration commonly requires a large internal data storage unit, such as an SD card.

**Figure 9:**
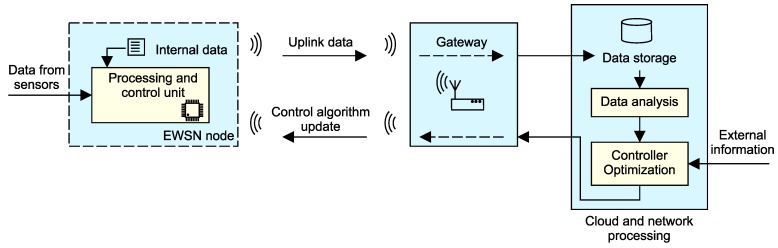
Possibilities to control an EWSN node through a downlink data channel.

Another research topic concerns the possibilities of dynamic sensor network control through a downlink communication channel, as shown in Figure 9.

The usage of a downlink communication channel enables the implementation of algorithms for updating the nodes’ operating parameters. This connection link allows the node to actively cooperate on specific monitoring tasks. There is also a possibility to predict the operating parameters at the cloud level and then cascade them down to the nodes. Data processing and prediction at the cloud level plays an important role in this operational paradigm. In many communication interfaces, a downlink is limited to few bytes per day, which makes it an interesting research area aiming for the development of data-effective control algorithms.

### 5.3. Future Trends

Future trends in the area of energy harvesting include the discovery and exploitation of unconventional energy sources [86]. Examples include harvesting energy from the water/soil pH difference [87,88], or from the movement of tree leaves [89] and trunks [90]. Indeed, many such approaches are well-suited for applications in EWSN, as they take advantage of phenomena which are commonly present in natural environments.

The field of energy storage is dominated by the search for batteries with high energy density as well as improved longevity and safety. In the case of storage devices for EWSN, an additional requirement is the minimization of their potential environmental impact. A prime example of an immature technology is the lithium–air battery [91,92] that promises great improvements in storage efficiency and longevity.

State-of-the-art EWSN devices use static operational strategies preset during network installation. Current research focuses on dynamic control and online energy management strategies [93]. These approaches allow the optimization of EWSN operation strategies at the level of individual nodes. They are capable, for example, of effectively dealing with the time-variant distribution of available energy [2].

The distributed network character of modern EWSNs provides additional opportunities for the minimization of energy consumption. One example is the use of clustering and zoning to design energy-aware data collection protocols [94]. The pinnacle of the current communication technology development is the realization of software-defined sensor networks (SDSNs). Not only are SDSNs able to adapt to specific application requirements, but they can also fully exploit the network resources, including energy. Zeng et al. [95] investigate three mechanisms to achieve globally optimized network energy efficiency: sensor activation, task mapping, and sensing scheduling.

## 6. Conclusions

This paper identifies and describes opportunities for future research in the area of environmental wireless sensor networks (EWSNs), which are typically deployed in remote locations without the possibility of frequent maintenance. Additionally, the paper provides an overview of state-of-the-art technologies that can be integrated into EWSNs so as to improve their reliability and operational efficiency.

One of the research challenges from a single node perspective is selection of energy management-related parameters which have direct impact on the utilization of energy harvesting sources and energy storage. Their optimization would increase the system reliability. Since there is always a trade-off between environmental data measurement rate and data transmission rate, the application of advanced control algorithms, such as fuzzy logic controller or reinforcement learning (both introduced in our previous studies), is an interesting research challenge and outlines one of the directions for our future work.

On the network level, one of the main incentives is to reduce the amount of data sent in both uplink and downlink directions via a network, which typically has some limitation in terms of data throughput per specific window of time. Therefore, the development of such solutions represents one of the most important research challenges.

Yet another research challenge could be the development of methods for data processing and the prediction of EWSN operating parameters at the cloud level. This data could then be cascaded down to sensor nodes through the downlink communication.

Since one of the weak points of every EWSN is its power system, the selection of hardware components suitable for a particular deployment environment is crucial. This paper reviews current solutions and new trends in the area of energy harvesting sources and alternatives for energy storage, where one of the directions for further development might be using components that incorporate new materials (e.g., graphene-based materials), which could greatly increase their performance.

Our future work will focus on addressing the research challenges mentioned above so as to arrive at a better EWSN design, which will help to collect good-quality environmental data.

## Figures and Tables

**Table 1:** Comparison of energy harvesting sources [38]. RF: radio frequency.

Power Source	Type	Power Density	Embedded Nominal Power	Output	Transducer
Wind	Mechanical	28.5 mW/cm${}^{2}$ [38],	47 dBm (50 W)	-	Wind Turbine
		$3.8\times 10^{-4}$ W/cm${}^{3}$ [13]			
Solar	Electromagnetic	15 mW/cm${}^{2}$ [38]	42 dBm (15 W)	0.5–1.0 V [33]	Solar Panels
Thermal	Thermal	15 μW/cm${}^{3}$ [38],	22 dBm (150 mW)	-	Thermoelectric Generator
		20–60 μW/cm${}^{2}$ [33],			
		40 μW/cm${}^{3}$ [13]			
Ambient RF	Electromagnetic	12 nW/cm${}^{2}$ [38],	−23 dBm (5 μW)	3–4 V [33]	Antenna
		0.2 mW/cm${}^{2}$–1 μW/cm${}^{2}$ [33]			

**Table 2:** Basic parameters of selected battery types [42,43,44,45,46,47].

Type	Rated Voltage (V)	Capacity (Ah)	Temperature Range (${}^{\circ }$C)	Cycling Capacity (-)	Specific Energy (Wh/kg)
Lead-Acid	2	1.3	−20–60	500–1000	30–50
MnO${}_{2}$Li	3	0.03–5	−20–60	1000–2000	280
Li poly-carbon	3	0.025–5	−20–60	-	100–250
LiSOCl${}_{2}$	3.6	0.025–40	−40–85	-	350
LiO${}_{2}$S	3	0.025–40	−60–85	-	500–700
NiCd	1.2	1.1	−40–70	10,000–20,000	50–60
NiMH	1.2	2.5	−20–40	1000–20,000	60–70
Li-Ion	3.6	0.74	−30–45	1000–100,000	75–200
MnO${}_{2}$	1.65	0.617	−20–60	-	300–610

**Table 3:** Basic parameters of selected supercapacitors (SCs) [11,51].

Supercapacitor	Life Cycle (-)	Specify Energy (Wh/kg)	Operating Temperature (${}^{\circ }$C)	Cell Voltage (V)
Maxwell PC10	500,000	1.4	−40–70	2.50
Maxwell BCAP0350	500,000	5.1	−40–70	2.50
Green-cap EDLC	>100,000	1.47	−40–60	2.70
EDLC SC	1,000,000	3–5	−40–65	2.70
Pseudo SC	100,000	10	−40–65	2.3–2.8
Hybrid SC	500,000	180	−40–65	2.3–2.8

**Table 4:** State-of-charge (SOC) estimation methods [56,58,59]. SOH: state-of-health.

SOC Technique	Field of Application	Field of Application	Field of Application
Coulomb Counter	All energy storage systems, most applications	Online, easy, accurate if enough re-calibration points are available and with good current measurement	Needs model for losses. Sensitive to parasite reactions. Cost-intensive for accurate measurement.
Open Circuit Voltage	Lead, lithium, Zn/Br, Va, and supercapacitors	Online, cheap	Low dynamic, problem of parasite reaction
Impedance Spectroscopy	All energy storage systems	Give information about SOH and quality. Possibility of online measurement	Temperature-sensitive, cost-intensive
Discharge Test	All energy storage systems. Used for capacity determination in the beginning of life	Easy and accurate, independent of SOH.	Offline, time-intensive, modifies the battery state, loss of energy

## Appendix A. Taxonomy of References Covered in This Review

**Table A1:** Categorization of reviewed articles.

Category	Relevant References
Batteries	[11,14,25,39,40,41,42,43,44,45,46,47,48,49,53,54,61,62,63,71,79,91,92,93]
Energy conversion	[22,23,29,30,33,36,38,42,43,46,55,69,75,76,78]
Environmental monitoring	[2,4,20,31,67,81,82,83,96]
Internet-of-Things	[22,34,97,98]
Networking	[33,34,37,68,70,74,83,85,93,94,95,97,98,99]
Power management	[2,3,4,10,13,16,17,21,30,31,32,40,67,68,69,70,71,72,73,76,77,78,79,80,81,82]
RF-based energy harvesting	[5,11,12,25,33,35,36,37,38,74,79]
Solar energy harvesting	[4,5,7,8,9,10,11,12,13,14,15,16,17,19,20,21,22,23,25,32,33,38,44,55,69,71,73,74,76,81,82]
Supercapacitor	[11,23,25,38,40,41,42,44,46,48,49,50,51,52,53,54,55,61,64,65,66,79]
State-of-charge	[56,57,58,59,60]
Thermal energy harvesting	[5,7,11,12,19,24,25,26,33,34,38]
Wind-based energy harvesting	[5,7,11,12,14,19,25,27,28,29,30,31,32,38,73]
Alternative harvesting approaches	[87,88,89,90]

## References

[1] Musilek P., Prauzek M., Kromer P., Rodway J., Barton T. (2017). Intelligent Energy Management for Environmental Monitoring Systems. Smart Sens. Netw..

[2] Prauzek M., Kromer P., Rodway J., Musilek P. (2016). Differential evolution of fuzzy controller for environmentally-powered wireless sensors. Appl. Soft Comput. J..

[3] Sundaran K., Murugaanandam S., Ganapathy V. (2016). Energy efficient techniques in wireless sensor networks: Recent survey. Sens. Lett..

[4] Prauzek M., Musilek P., Watts A. Fuzzy algorithm for intelligent wireless sensors with solar harvesting. Proceedings of the 2014 IEEE Symposium Series on Computational Intelligence.

[5] Shaikh F.K., Zeadally S. (2016). Energy harvesting in wireless sensor networks: A comprehensive review. Renew. Sustain. Energy Rev..

[6] Jiao P., Borchani W., Hasni H., Lajnef N. (2017). Enhancement of quasi-static strain energy harvesters using non-uniform cross-section post-buckled beams. Smart Mater. Struct..

[7] Shad R., Steingart D., Frechette L., Wright P., Rabaey J., Karl H., Wolisz A., Willig A. (2004). Power Sources for Wireless Sensor Networks. Wireless Sensor Networks.

[8] Mousavi S.M., Mostafavi E.S., Jiao P. (2017). Next generation prediction model for daily solar radiation on horizontal surface using a hybrid neural network and simulated annealing method. Energy Convers. Manag..

[9] Milichko V.A., Shalin A.S., Mukhin I.S., Kovrov A.E., Krasilin A.A., Vinogradov A.V., Belov P.A., Simovski C.R. (2016). Solar photovoltaics: Current state and trends. Physics-Uspekhi.

[10] Mungan E.S., Lu C., Raghunathan V., Roy K. Modeling, Design and Cross-layer Optimization of Polysilicon Solar Cell Based Micro-scale Energy Harvesting Systems. Proceedings of the 2012 ACM/IEEE International Symposium on Low Power Electronics and Design.

[11] Akhtar F., Rehmani M.H. (2015). Energy replenishment using renewable and traditional energy resources for sustainable wireless sensor networks: A review. Renew. Sustain. Energy Rev..

[12] Panatik K., Kamardin K., Shariff S., Yuhaniz S., Ahmad N., Yusop O., Ismail S. Energy harvesting in wireless sensor networks: A survey. Proceedings of the 2016 IEEE 3rd International Symposium on Telecommunication Technologies.

[13] Raghunathan V., Kansal A., Hsu J., Friedman J., Srivastava M. Design considerations for solar energy harvesting wireless embedded systems. Proceedings of the 2005 4th International Symposium on Information Processing in Sensor Networks.

[14] Sudevalayam S., Kulkarni P. (2011). Energy Harvesting Sensor Nodes: Survey and Implications. IEEE Commun. Surv. Tutor..

[15] Kelly N.A., Gibson T.L. (2011). Increasing the solar photovoltaic energy capture on sunny and cloudy days. Sol. Energy.

[16] Shao H., Tsui C.Y., Ki W.H. A micro power management system and maximum output power control for solar energy harvesting applications. Proceedings of the 2007 ACM/IEEE International Symposium on Low Power Electronics and Design (ISLPED).

[17] Shao H., Tsui C.Y., Ki W.H. An inductor-less micro solar power management system design for energy harvesting applications. Proceedings of the 2007 IEEE International Symposium on Circuits and Systems.

[18] Bardwell M., Wong J., Zhang S., Musilek P. Design Considerations for IoT-based PV Charge Controllers. Proceedings of the IEEE World Congress on Services.

[19] Zhou G., Huang L., Li W., Zhu Z. (2014). Harvesting ambient environmental energy for wireless sensor networks: A survey. J. Sens..

[20] Zou T., Lin S., Feng Q., Chen Y. (2016). Energy-efficient control with harvesting predictions for solar-powered wireless sensor networks. Sensors.

[21] Buchli B., Sutton F., Beutel J., Thiele L. (2014). Towards Enabling Uninterrupted Long-Term Operation of Solar Energy Harvesting Embedded Systems. Proceedings of the 11th European Conference on Wireless Sensor Networks.

[22] Li Y., Shi R. (2015). An intelligent solar energy-harvesting system for wireless sensor networks. EURASIP J. Wirel. Commun. Netw..

[23] Kim S., Chou P.H. Energy harvesting by sweeping voltage-escalated charging of a reconfigurable supercapacitor array. Proceedings of the 17th IEEE/ACM International Symposium on Low-Power Electronics and Design.

[24] Nguyen N.Q., Pochiraju K.V. (2013). Behavior of thermoelectric generators exposed to transient heat sources. Appl. Therm. Eng..

[25] Penella-López M.T., Gasulla-Forner M. (2011). Powering Autonomous Sensors.

[26] Datta U., Dessouky S., Papagiannakis A. (2017). Harvesting thermoelectric energy from asphalt pavements. Transp. Res. Rec..

[27] Mendonça F., Azevedo J. Design and power production of small-scale wind turbines. Proceedings of the 2017 International Conference in Energy and Sustainability in Small Developing Economies (ES2DE).

[28] Pimentel D., Musilek P., Knight A., Heckenbergerova J. Characterization of a wind flutter generator. Proceedings of the 2010 9th Conference on Environment and Electrical Engineering.

[29] Carli D., Brunelli D., Bertozzi D., Benini L. A high-efficiency wind-flow energy harvester using micro turbine. Proceedings of the 2010 International Symposium on Power Electronics Electrical Drives Automation and Motion (SPEEDAM).

[30] Tan Y.K., Panda S.K. (2011). Optimized wind energy harvesting system using resistance emulator and active rectifier for wireless sensor nodes. IEEE Trans. Power Electron..

[31] Jushi A., Pegatoquet A., Le T.N. Wind Energy Harvesting for Autonomous Wireless Sensor Networks. Proceedings of the 2016 Euromicro Conference on Digital System Design (DSD).

[32] Baranov A., Spirjakin D., Akbari S., Somov A., Passerone R. (2016). POCO: ‘Perpetual’ operation of CO wireless sensor node with hybrid power supply. Sens. Actuators A Phys..

[33] Kim S., Vyas R., Bito J., Niotaki K., Collado A., Georgiadis A., Tentzeris M. (2014). Ambient RF energy-harvesting technologies for self-sustainable standalone wireless sensor platforms. Proc. IEEE.

[34] Kamalinejad P., Mahapatra C., Sheng Z., Mirabbasi S., Leung V., Guan Y. (2015). Wireless Energy Harvesting for the Internet of Things. IEEE Commun. Mag..

[35] Dinesh Kumar K., Hemalatha M. (2014). Designing EM energy harvesting antenna to give power support to embedded sensor. Int. J. Appl. Eng. Res..

[36] Lorenz C., Hemour S., Li W., Xie Y., Gauthier J., Fay P., Wu K. (2015). Breaking the Efficiency Barrier for Ambient Microwave Power Harvesting with Heterojunction Backward Tunnel Diodes. IEEE Trans. Microw. Theory Tech..

[37] Vyas R., Cook B., Kawahara Y., Tentzeris M. (2013). E-WEHP: A batteryless embedded sensor-platform wirelessly powered from ambient digital-TV signals. IEEE Trans. Microw. Theory Tech..

[38] Habibzadeh M., Hassanalieragh M., Ishikawa A., Soyata T., Sharma G. (2017). Hybrid Solar-Wind Energy Harvesting for Embedded Applications: Supercapacitor-Based System Architectures and Design Tradeoffs. IEEE Circuits Syst. Mag..

[39] Ertugrul N. Battery storage technologies, applications and trend in renewable energy. Proceedings of the 2016 IEEE International Conference on Sustainable Energy Technologies (ICSET).

[40] Tan X., Li Q., Wang H. (2013). Advances and trends of energy storage technology in Microgrid. Int. J. Electr. Power Energy Syst..

[41] Sullivan J., Gaines L. (2010). A Review of Battery Life-Cycle Analysis: State of Knowledge and Critical Needs.

[42] Aneke M., Wang M. (2016). Energy storage technologies and real life applications–A state of the art review. Appl. Energy.

[43] Zhang C., Wei Y.L., Cao P.F., Lin M.C. (2017). Energy storage system: Current studies on batteries and power condition system. Renew. Sustain. Energy Rev..

[44] Taneja J., Jeong J., Culler D. (2008). Design, modeling, and capacity planning for micro-solar power sensor networks. Proceedings of the 7th International Conference on Information Processing in Sensor Networks.

[45] Yadav G.G., Wei X., Huang J., Turney D., Nyce M., Banerjee S. (2018). Accessing the second electron capacity of MnO2 by exploring complexation and intercalation reactions in energy dense alkaline batteries. Int. J. Hydrog. Energy.

[46] Akinyele D., Rayudu R. (2014). Review of energy storage technologies for sustainable power networks. Sustain. Energy Technol. Assess..

[47] Kaldellis J., Zafirakis D. (2007). Optimum energy storage techniques for the improvement of renewable energy sources-based electricity generation economic efficiency. Energy.

[48] Zou C., Zhang L., Hu X., Wang Z., Wik T., Pecht M. (2018). A review of fractional-order techniques applied to lithium-ion batteries, lead-acid batteries, and supercapacitors. J. Power Sources.

[49] Bradbury K. (2010). Energy Storage Technology Review.

[50] Okonkwo P., Collins E., Okonkwo E. (2017). Application of Biopolymer Composites in Super Capacitor. Biopolymer Composites in Electronics.

[51] Libich J., Máca J., Vondrák J., Čech O., Sedlaříková M. (2018). Supercapacitors: Properties and applications. J. Energy Storage.

[52] Merrett G., Weddell A. Supercapacitor leakage in energy-harvesting sensor nodes: Fact or fiction?. Proceedings of the 9th International Conference on Networked Sensing Systems.

[53] Renner C., Jessen J., Turau V. Lifetime prediction for supercapacitor-powered wireless sensor nodes. Proceedings of the GI/ITG Fachgespräch “Sensornetze” (FGSN’09).

[54] Luo X., Wang J., Dooner M., Clarke J. (2015). Overview of current development in electrical energy storage technologies and the application potential in power system operation. Appl. Energy.

[55] Simjee F.I., Chou P.H. (2008). Efficient charging of supercapacitors for extended lifetime of wireless sensor nodes. IEEE Trans. Power Electr..

[56] Piller S., Perrin M., Jossen A. (2001). Methods for state-of-charge determination and their applications. J. Power Sources.

[57] Ng K., Moo C.S., Chen Y.P., Hsieh Y.C. (2009). Enhanced coulomb counting method for estimating state-of-charge and state-of-health of lithium-ion batteries. Appl. Energy.

[58] Kalawoun J., Biletska K., Suard F., Montaru M. (2015). From a novel classification of the battery state of charge estimators toward a conception of an ideal one. J. Power Sources.

[59] Pop V., Bergveld H., Notten P., Regtien P. (2005). State-of-the-art of battery state-of-charge determination. Meas. Sci. Technol..

[60] Coleman M., Lee C., Zhu C., Hurley W. (2007). State-of-charge determination from EMF voltage estimation: Using impedance, terminal voltage, and current for lead-acid and lithium-ion batteries. IEEE Trans. Ind. Electr..

[61] Lv W., Li Z., Deng Y., Yang Q.H., Kang F. (2016). Graphene-based materials for electrochemical energy storage devices: Opportunities and challenges. Energy Storage Mater..

[62] Song J., Xu T., Gordin M.L., Zhu P., Lv D., Jiang Y.B., Chen Y., Duan Y., Wang D. (2014). Nitrogen-Doped Mesoporous Carbon Promoted Chemical Adsorption of Sulfur and Fabrication of High-Areal-Capacity Sulfur Cathode with Exceptional Cycling Stability for Lithium-Sulfur Batteries. Adv. Funct. Mater..

[63] Li W., Yang Z., Li M., Jiang Y., Wei X., Zhong X., Gu L., Yu Y. (2016). Amorphous red phosphorus embedded in highly ordered mesoporous carbon with superior lithium and sodium storage capacity. Nano Lett..

[64] Bichat M., Raymundo-Piñero E., Béguin F. (2010). High voltage supercapacitor built with seaweed carbons in neutral aqueous electrolyte. Carbon.

[65] Newell R., Faure-Vincent J., Iliev B., Schubert T., Aradilla D. (2018). A new high performance ionic liquid mixture electrolyte for large temperature range supercapacitor applications (−70 °C to 80 °C) operating at 3.5 V cell voltage. Electrochim. Acta.

[66] Khomenko V., Raymundo-Pinero E., Frackowiak E., Beguin F. (2006). High-voltage asymmetric supercapacitors operating in aqueous electrolyte. Appl. Phys. A.

[67] Kansal A., Potter D., Srivastava M. (2004). Performance aware tasking for environmentally powered sensor networks. Perform. Eval. Rev..

[68] Vigorito C., Ganesan D., Barto A. Adaptive control of duty cycling in energy-harvesting wireless sensor networks. Proceedings of the 2007 4th Annual IEEE Communications Society Conference on Sensor, Mesh and Ad Hoc Communications and Networks.

[69] Hsu J., Zahedi S., Kansal A., Srivastava M., Raghunathan V. Adaptive duty cycling for energy harvesting systems. Proceedings of the International Symposium on Low Power Electronics and Design.

[70] Kansal A., Hsu J., Srivastava M., Raghunathan V. Harvesting aware power management for sensor networks. Proceedings of the 43rd Annual Design Automation Conference.

[71] Raghunathan V., Chou P.H. Design and Power Management of Energy Harvesting Embedded Systems. Proceedings of the 2006 International Symposium on Low Power Electronics and Design.

[72] Kansal A., Hsu J., Zahedi S., Srivastava M.B. (2007). Power management in energy harvesting sensor networks. ACM Trans. Embed. Comput. Syst..

[73] Pimentel D., Musilek P. Power management with energy harvesting devices. Proceedings of the 2010 23rd Canadian Conference on Electrical and Computer Engineering (CCECE).

[74] Pirapaharan K., Gunathillake W., Lokunarangoda G., Nissansani M., Palihena H., Hoole P., Aravind C., Hoole S. Design of a battery-less micro-scale RF energy harvester for medical devices. Proceedings of the 2012 IEEE-EMBS Conference on Biomedical Engineering and Sciences.

[75] Yi J., Su F., Lam Y.H., Ki W.H., Tsui C.Y. An energy-adaptive MPPT power management unit for micro-power vibration energy harvesting. Proceedings of the IEEE International Symposium on Circuits and Systems.

[76] Bergonzini C., Brunelli D., Benini L. Algorithms for harvested energy prediction in batteryless wireless sensor networks. Proceedings of the 3rd International Workshop on Advances in Sensors and Interfaces.

[77] Liu S., Lu J., Wu Q., Qiu Q. (2012). Harvesting-aware power management for real-time systems with renewable energy. IEEE Trans. Very Large Scale Integr. (VLSI) Syst..

[78] Lu C., Park S.P., Raghunathan V., Roy K. Stage number optimization for switched capacitor power converters in micro-scale energy harvesting. Proceedings of the 2011 Design, Automation & Test in Europe.

[79] Lu C., Raghunathan V., Roy K. (2011). Efficient design of micro-scale energy harvesting systems. IEEE J. Emerg. Sel. Top. Circuits Syst..

[80] Janek A., Steger C., Preishuber-Pfluegl J., Pistauer M. Power management strategies for battery-driven higher Class UHF RFID tags supported by energy harvesting devices. Proceedings of the 2007 IEEE Workshop on Automatic Identification Advanced Technologies.

[81] Watts A., Prauzek M., Musilek P., Pelikan E., Sanchez-Azofeifa A. Fuzzy power management for environmental monitoring systems in tropical regions. Proceedings of the International Joint Conference on Neural Networks.

[82] Prauzek M., Musilek P., Watts A., Michalikova M. (2014). Powering environmental monitoring systems in arctic regions: A simulation study. Elektron. Elektrotech..

[83] Krömer P., Prauzek M., Musilek P., Barton T. (2014). Optimization of Wireless Sensor Node Parameters by Differential Evolution and Particle Swarm Optimization. Adv. Intell. Syst. Comput..

[84] Prauzek M., Mourcet M., Hlavica J., Musilek P. Q-learning Algorithm for Energy Management in Solar Powered Embedded Monitoring Systems. Proceedings of the 2018 IEEE Congress on Evolutionary Computation.

[85] Pötsch A., Haslhofer F. Practical limitations for deployment of LoRa gateways. Proceedings of the 2017 IEEE International Workshop on Measurement and Networking.

[86] Lee D., Dulai G., Karanassios V. (2013). Survey of energy harvesting and energy scavenging approaches for on-site powering of wireless sensor- and microinstrument-networks. Proc. SPIE.

[87] Bhuyan S., Hu J. (2013). A natural battery based on lake water and its soil bank. Energy.

[88] Trizcinski P., Nathan A., Karanassios V. (2017). Approaches to energy harvesting and energy scavenging for energy autonomous sensors and microinstruments. Proc. SPIE.

[89] McGarry S., Knight C. (2011). The potential for harvesting energy from the movement of trees. Sensors.

[90] McGarry S., Knight C. (2012). Development and successful application of a tree movement energy harvesting device, to power a wireless sensor node. Sensors.

[91] Asadi M., Sayahpour B., Abbasi P., Ngo A.T., Karis K., Jokisaari J.R., Liu C., Narayanan B., Gerard M., Yasaei P. (2018). A lithium-oxygen battery with a long cycle life in an air-like atmosphere. Nature.

[92] Wu F., Yu Y. (2018). Toward True Lithium-Air Batteries. Joule.

[93] Ulukus S., Yener A., Erkip E., Simeone O., Zorzi M., Grover P., Huang K. (2015). Energy harvesting wireless communications: A review of recent advances. IEEE J. Sel. Areas Commun..

[94] Gallegos A., Noguchi T., Izumi T., Nakatani Y. (2018). Zone-based energy aware data collection protocol for WSNs. IEICE Trans. Commun..

[95] Zeng D., Li P., Guo S., Miyazaki T., Hu J., Xiang Y. (2015). Energy Minimization in Multi-Task Software-Defined Sensor Networks. IEEE Trans. Comput..

[96] Ahmad M., Mourshed M., Mundow D., Sisinni M., Rezgui Y. (2016). Building energy metering and environmental monitoring—A state-of-the-art review and directions for future research. Energy Build..

[97] Raza U., Kulkarni P., Sooriyabandara M. (2017). Low Power Wide Area Networks: An Overview. IEEE Commun. Surv. Tutor..

[98] Yue Y., He P. (2018). A comprehensive survey on the reliability of mobile wireless sensor networks: Taxonomy, challenges, and future directions. Inf. Fusion.

[99] Yick J., Mukherjee B., Ghosal D. (2008). Wireless sensor network survey. Comput. Netw..

